# A novel biplanar medial opening-wedge high tibial osteotomy: the Z-shaped technique. A case series at 7.2 years follow-up

**DOI:** 10.1186/s10195-021-00617-4

**Published:** 2021-12-14

**Authors:** Marcello Presutti, Remo Goderecci, Pietro Palumbo, Alessio Giannetti, Manuel Giovanni Mazzoleni, Filippo Maria Nicola Randelli, Massimo Angelozzi, Vittorio Calvisi, Andrea Fidanza

**Affiliations:** 1 Unit of Orthopaedic Surgery, “Casa Di Cura Villa Esther”, Bojano (Cb), Italy; 2 Unit of Orthopaedics and Traumatology, “G. Mazzini” Civil Hospital of Teramo, ASL 4 Teramo, Piazza Italia 1, 64100 Teramo, Italy; 3 Unit of Orthopaedics and Traumatology, Azienda Ospedaliero Universitaria - Ospedali Riuniti, Foggia (FG), Italy; 4 Department of Life, Health and Environmental Sciences, Unit of Orthopaedics and Traumatology, University of L’Aquila, Piazzale Salvatore Tommasi 1, Blocco 11, 67100 L’Aquila (AQ), Italy; 5 Hip Department (CAD) Gaetano Pini - CTO Orthopedic Institute, University of Milan, Milan (MI), Italy

**Keywords:** Osteoarthritis, Unicompartmental knee osteoarthritis, High tibial osteotomy, HTO, Medial opening-wedge high tibial osteotomy, MOWHTO, Modified biplanar osteotomy, Z-shaped osteotomy, Puddu plate, Knee

## Abstract

**Background:**

High tibial osteotomy (HTO) provides reliable and good long-term results, if performed with correct indications, but different techniques and types of fixation have been described. The purpose of this study is to present a novel modified biplanar medial opening-wedge (MOW) HTO technique where the osteotomies are performed in a Z-shaped fashion, and to present the medium-term clinical and radiographic results.

**Materials and methods:**

We present a case series of 75 patients (80 knees) with mean age of 45.8 years, affected by isolated medial knee osteoarthritis and symptomatic varus knee malalignment, who underwent novel biplanar Z-shaped MOWHTO. Clinical and radiological outcomes were collected, retrospectively before surgery and at median follow-up of 7.2 years (95% CI 5.6–9.2 months) after surgery. Clinical results and satisfaction were assessed by visual analog scale (VAS), Western Ontario and McMaster University Osteoarthritis Index (WOMAC), and Likert scale. Radiological assessment involved the evaluation of the medial proximal tibial angle (MPTA), tibial slope (TS), Caton–Deschamps index, and knee osteoarthritis grade according to Ahlbäck classification. Pre- and postoperative results were compared using the two-tailed *t*-test or Wilcoxon’s test of independent samples for paired data or nonparametric analog. *P* < 0.05 was considered significant.

**Results:**

At medium-term follow-up, Z-shaped MOWHTO showed a survival rate of 95 ± 1.7% with failure occurring in four knees due to symptom recurrence and osteoarthritis progression. No perioperative complications were observed (intraarticular fracture, delayed union or nonunion, and neurological injury). Mean bone healing time was 12 weeks. Clinical scores showed significant improvement at last follow-up and a good grade of satisfaction. MPTA increased significantly, while Caton–Deschamps index decreased significantly. No significant TS increase was found.

**Conclusions:**

Modified biplanar Z-shaped MOWHTO is a safe and reliable technique that offers satisfactory clinical and radiological medium-term outcomes with low knee arthroplasty conversion rate. The unique three-dimensional geometrical conformation potentially provides a favorable environment for bone healing, increased anteroposterior and rotational stability, and safer opening-wedge loading force application with low lateral hinge fracture risk.

**Level of evidence:**

Level IV, retrospective observational case series study.

*Trial registration* The study protocol was approved by the Internal Review Board of our Institution (authorization number 54/2019, 20 November 2019).

## Introduction

Knee osteoarthritis (OA) is a pervasive orthopedic pathology with significant socioeconomic burden in terms of direct and indirect costs [1].

The most common degenerative pattern involves primarily the medial compartment as a result of an unfavorable load transmission of varus knee deformity [2–5].

The first treatment line is conservative, mainly symptomatic, to delay joint replacement as long as possible [6]. Younger patients with mild OA tend to be dissatisfied after total knee arthroplasty (TKA) [7, 8], and unicompartmental knee arthroplasty (UKA), in case of medial OA in a varus knee of an active patients, is still discussed but controversial in literature [9, 10].

High tibial osteotomy (HTO) is a viable and cost-effective preserving surgery that allows pain reduction and return to physical activities [11, 12].

HTO is indicated in younger active but symptomatic patients (generally younger than 65 years old) affected by an arthritic medial compartment, with an axial deformity angle lower than 20° and a knee flexion range of at least 100° [13].

Different HTO strategies and types of fixation have been described [14]. Historically, this procedure was commonly performed by lateral closing-wedge HTO (LCWHTO) [15]. This technique still represents an effective treatment but is technically demanding and at higher risk of complications [13, 16]. LCWHTO is rarely performed nowadays, since the medial opening-wedge HTO (MOWHTO) technique was developed, and became popular, providing several advantages, such as easier and more accurate correction, with good long-term results [17–19]. Other advantages of MOWHTO include: conservation of the proximal tibial anatomy and bone stock which allows easier conversion to TKA; accurate protection of the peroneal nerve, avoiding fibular osteotomy; preservation of the proximal tibiofibular joint; and reduction of compartment syndrome risk [20, 21]. Nevertheless, MOWHTO has possible complications including delayed union, posterior slope increase, and patellar height reduction, and higher risk of lateral hinge or cortex fracture [4, 13, 22, 23]. MOWHTO was initially described as a single monoplanar osteotomy, oblique or transverse to the frontal plane [24, 25]. Lately, biplanar MOWHTO techniques have been described, such as the V-shaped or retrotubercle osteotomy [26–28]. Both of these techniques are currently those performed most frequently.

The smaller gap volume, and wider bone contact, are expected to promote faster and undisturbed bone ossification [29].

**Fig. 1:**
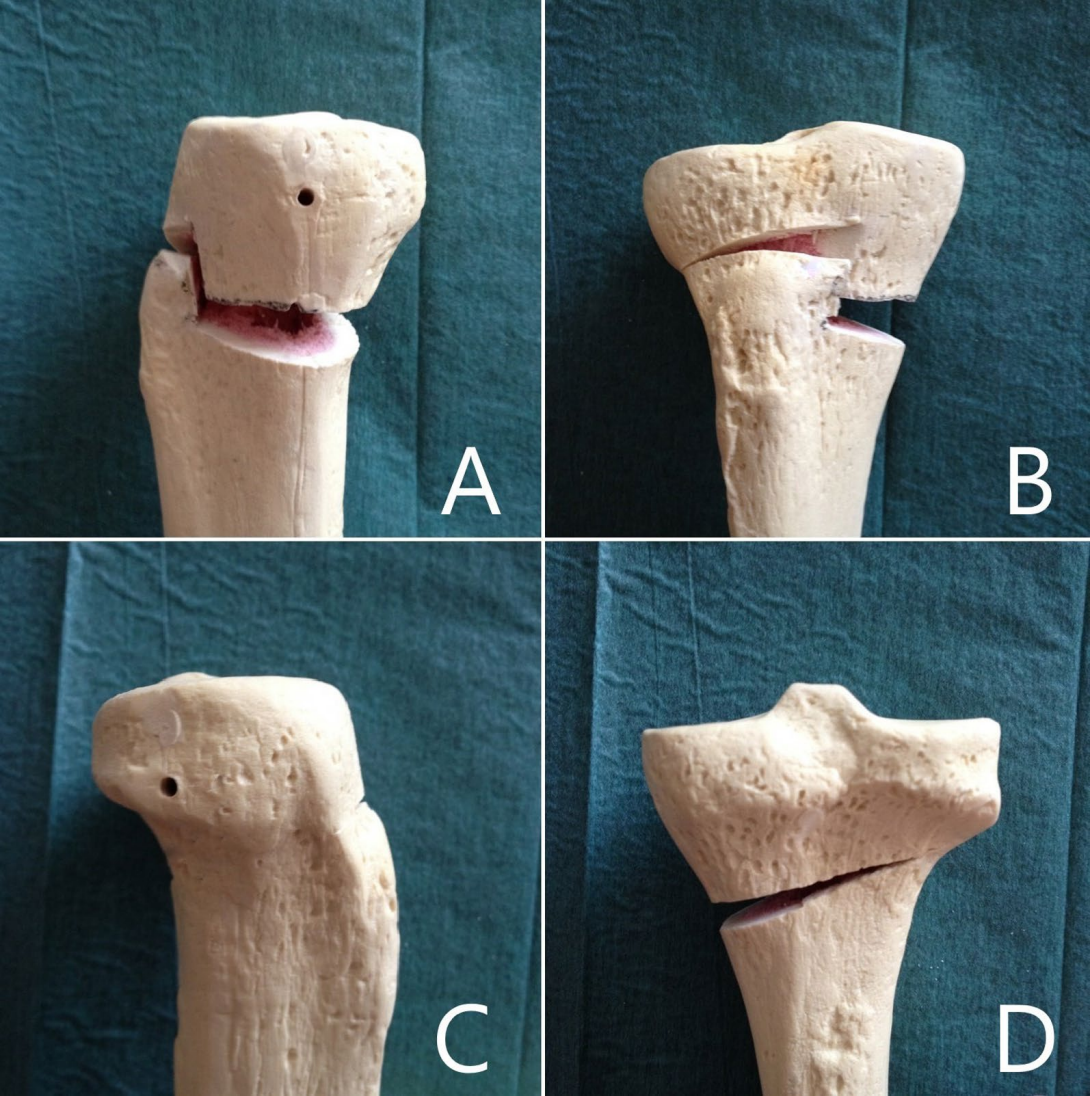
Reproduction of Z-shaped biplanar medial opening-wedge high tibia osteotomy technique on artificial bone model of left proximal tibial: **A** medial, **B** anterior, **C** lateral, and **D** posterior view

The goal of this study is to present a novel biplanar Z-shaped MOWHTO technique (Fig. 1) and its long-term clinical and radiological outcomes.

This new Z-shaped osteotomy conformation, compared with classic biplanar osteotomies, improves primary stability and firmness by widening the cancellous bone surfaces in contact and orienting one of the three osteotomies on a coronal plane, parallel to the tibial axis.

The hypothesis of the authors was that a biplanar Z-shaped MOWHTO can ensure high survival rates after long-term follow-up, with satisfactory functional and radiological results and low rate of complications.

## Materials and methods

We present a consecutive case series of 75 patients (80 knees) who underwent Z-shaped MOWHTO. All the procedures were performed from 2001 at a single orthopedic center (Casa di Cura Villa Esther in Bojano (Cb), Italy), by a single senior surgeon, who designed the surgical technique. To date, at median follow-up of 7.2 years (95% CI 5.6–9.2 months), patients have undergone clinical and radiological assessment. All data were collected prospectively and analyzed retrospectively by an independent assessor. All procedures were performed in accordance with ethical standards, and the study protocol was approved by the Internal Review Board of our Institution (authorization number 54/2019, 20 November 2019). All patients were informed and provided written informed consent to participate in the study. The study complied with current national and international laws and regulations governing the use of human subjects (Declaration of Helsinki and its later amendments).

The patients’ indications for undergoing Z-shaped MOWHTO were: knee pain associated with varus malalignment combined with isolated medial compartment osteoarthritis of the knee. We considered unsuitable for surgery those patients with active knee flexion lower than 120° or extension deficiency exceeding 10°. Contraindications for Z-shaped MOWHTO also included high-grade knee ligamentous instabilities, severe knee osteoarthritis (grade 4 and 5 according to Ahlbäck classification), active local or systemic infection, and/or inflammatory arthropathy. The study inclusion criteria were patients who have undergone Z-shaped MOWHTO since January 2001 with available preoperative and postoperative clinical and radiological data, recorded by the senior surgeon (M.P.) in his personal digital database (VisualMed by Efeso Active Marketing S.r.l). Exclusion criteria from the study were unavailability of preoperative or postoperative clinical or radiological dataset, and unwillingness to participate.

### Preoperative assessment

Preoperatively, the patients were investigated with a full-length standing anteroposterior radiograph of the entire lower extremities for evaluation of alignment of lower limbs. The knee joints were further investigated by bilateral radiographs in Rosenberg view, anteroposterior view, and true lateral view at 30° knee flexion [30]. The knee osteoarthritis grade was evaluated radiographically according to Ahlbäck classification as follows: grade 1, 30%; grade 2, 50.5%; grade 3, 19.5% [31].

### Surgical technique

The surgical procedure is performed with the patient in supine position on a radiolucent operating table and using a pneumatic tourniquet.

The center of the femoral head is identified preoperatively by fluoroscopy and marked with an adhesive metal reference on the inguinal skin. This reference can be palpated through the sterile draping and is used as a landmark for the alignment measurement rod. The center of the ankle and the knee are also determined by fluoroscopy.

After tourniquet inflation, diagnostic arthroscopy can be performed followed by meniscal and cartilaginous debridement, if indicated, at the medial compartment of the knee. Microfractures or chondroplasty can be performed as needed.

**Fig. 2:**
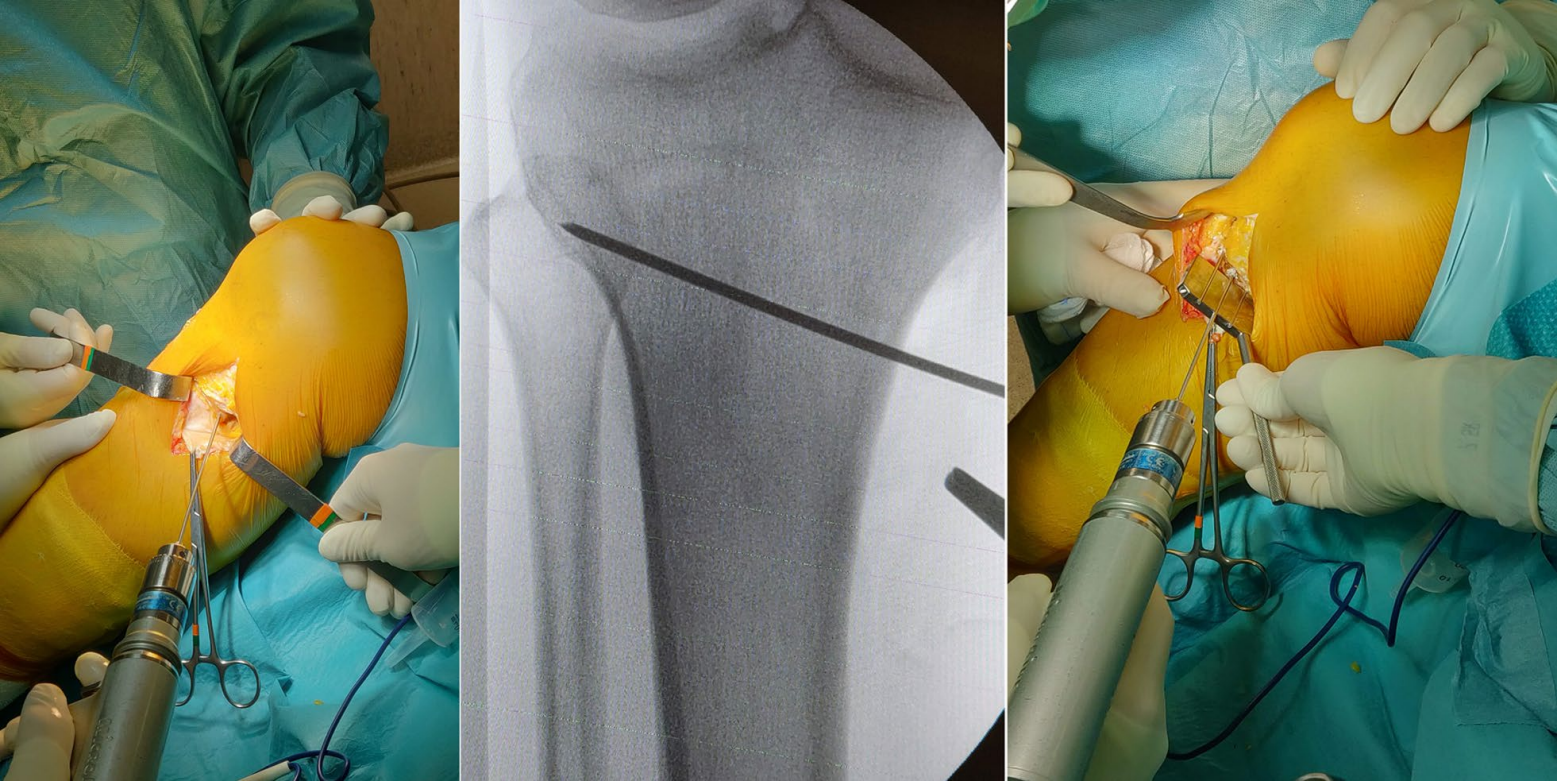
Intraoperative findings and fluoroscopic imaging of the first guide wire drilled through the proximal right tibia from medial to lateral directed 20° upwards. The first wire is advanced, under fluoroscopic guidance, toward the superior part of the fibular head. On the right is shown the positioning of the two additional parallel wires with the assistance of a self-made guiding device

After the first arthroscopic surgical time, the arthroscopic portals are sutured, and the operative field is prepared for the MOWHTO procedure. With the knee flexed at 90°, a 5–8-cm longitudinal skin incision is performed, centered approximately 4 cm distal to the medial joint line, midway between the posterior medial tibial crest and the tibial tuberosity. After superficial dissection, the underlying sartorial fascia is exposed, incised, and carefully reflected off the underlying gracilis and semitendinosus tendons, which can be identified and detached from their distal insertion. The distal portion of the medial collateral ligament is exposed and elevated from the medial tibial cortex as far as the level of the planned osteotomy. After exposure of the proximal tibia, a blunt Hohmann retractor is placed on the posterior aspect of the tibial metaphysis to elevate and protect the medial collateral ligament and neurovascular structures. This allows exposure of the posteromedial corner of the tibia and facilitates the final placement of the plate on a posteromedial position. A second retractor is placed under the patellar tendon. Under fluoroscopic control, a guide wire is drilled “freehand” from the proximal tibia, approximately 6 cm distal from the joint line, in a medial to lateral and upward direction (Fig. 2). The guide wire is directed approximately 20° upwards, aiming the superior part of the head of the fibula. Once the correct orientation of the first guide wire is obtained, under fluoroscopic guidance, two additional wires are drilled parallel to the first one, with the assistance of a first specific self-made guide (Fig. 2). The drilled wires are measured to calculate the exact length of the osteotomy.

**Fig. 3:**
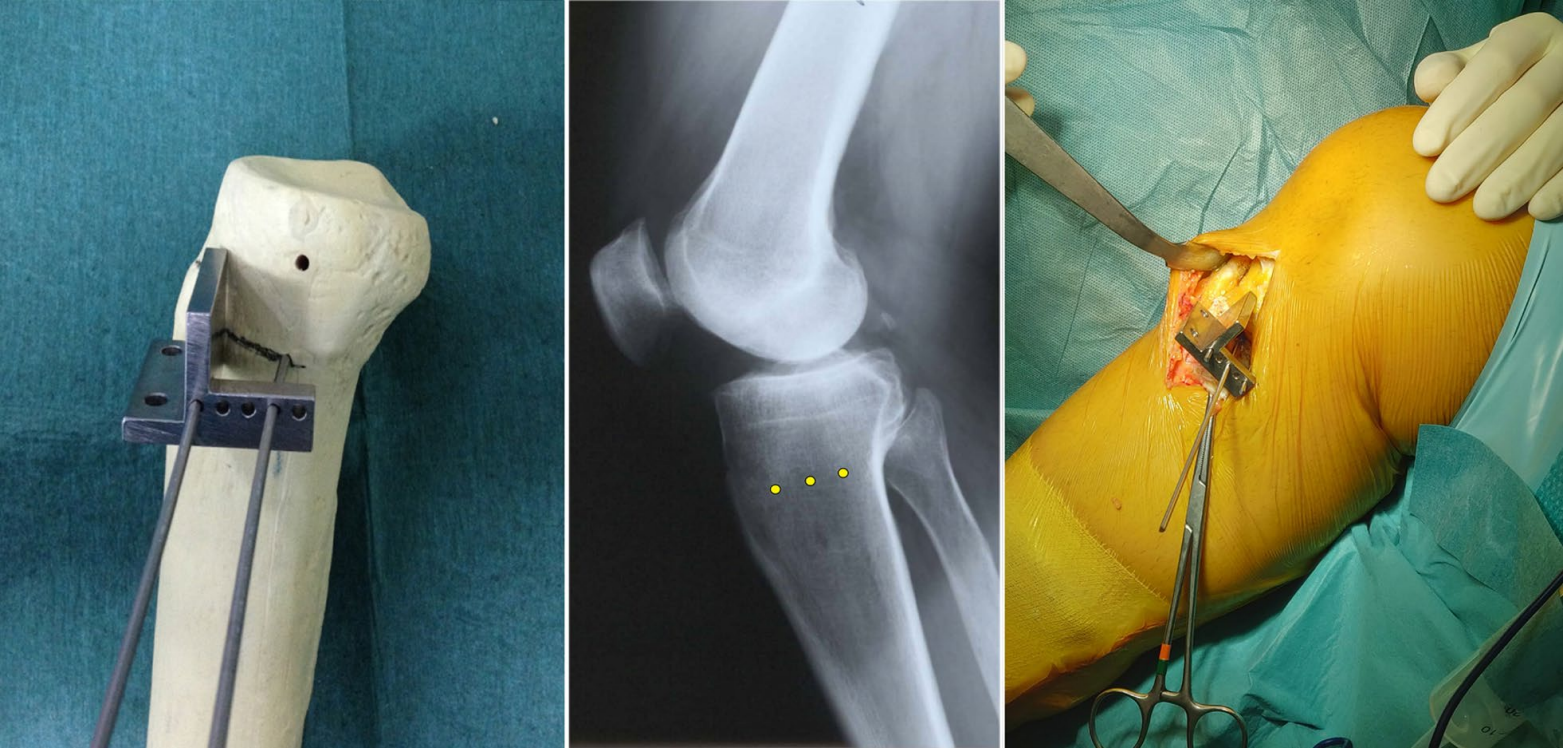
On the left, a demonstration of the second self-made cutting guide on a right tibia bone model. In the middle, a graphical representation of the three guide wires (yellow dots) on a lateral X-ray view of a right knee. On the right, the intraoperative findings of the self-made cutting guide positioning over the three wires, in a right tibia

**Fig. 4:**
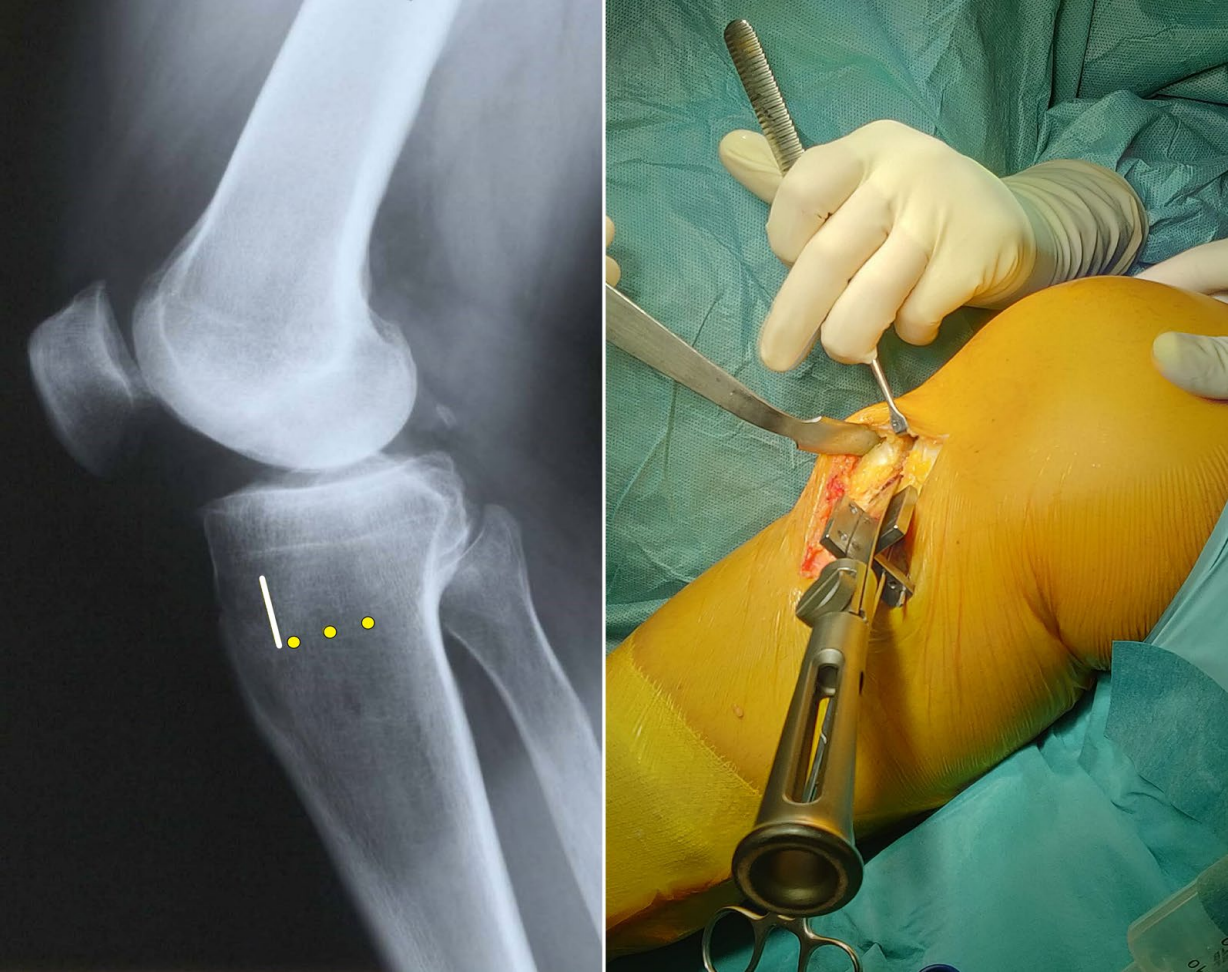
On the left, a graphical representation of the three guide wires (yellow dots) and the first coronal osteotomy (vertical white line), on a lateral X-ray view of a right knee. On the right, the intraoperative findings of the first coronal osteotomy completed with a sharp thin 2-cm large osteotome, in a right tibia, with the assistance of the self-made cutting guide

**Fig. 5:**
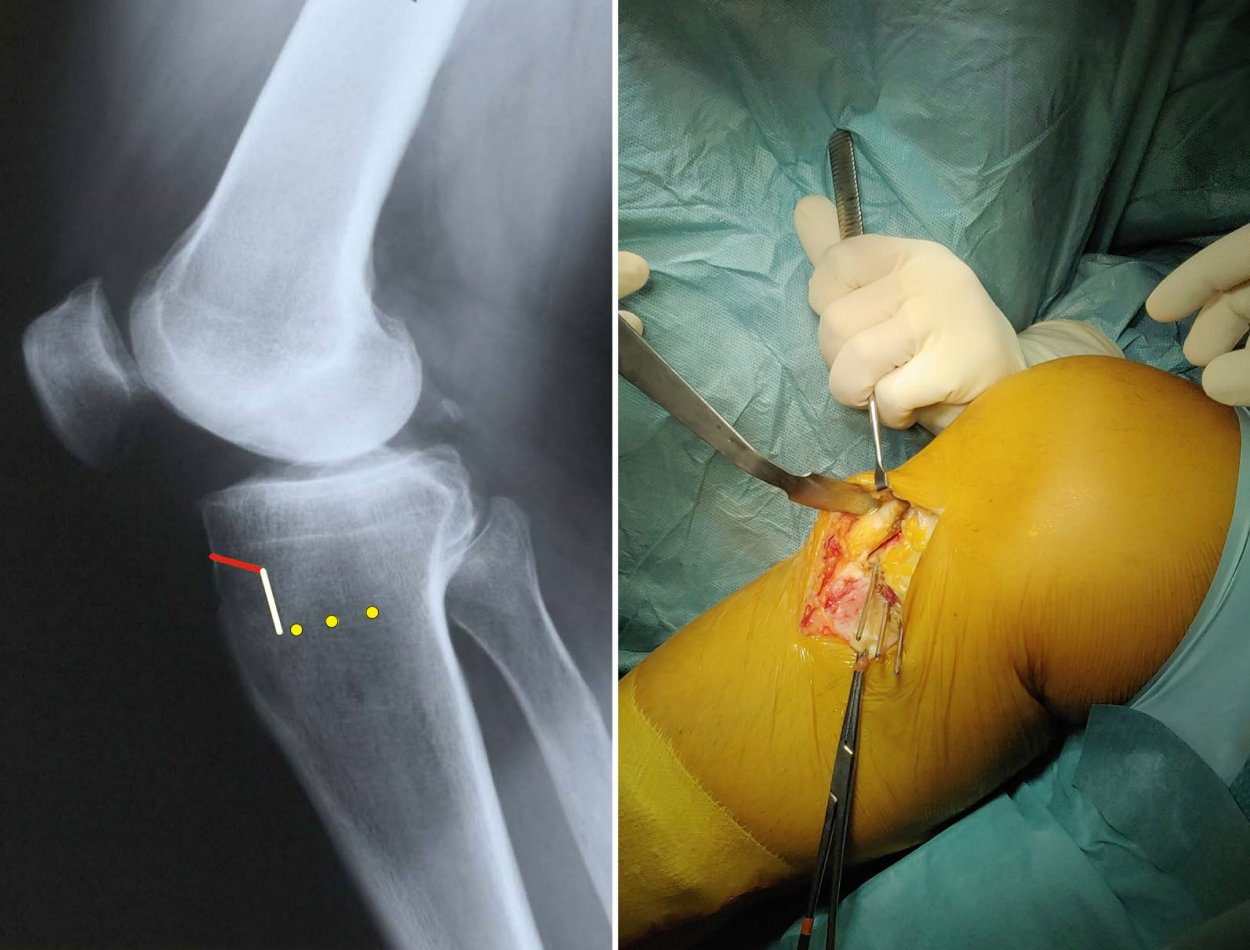
On the left, a graphical representation of the three guide wires (yellow dots), the first coronal osteotomy (vertical white line) and the second osteotomy (red line) on an oblique plane, on a lateral X-ray view of a right knee. On the right, the intraoperative findings of the first two osteotomies, in a right tibia. The three wires are still positioned to guide the next last osteotomy

Using a special self-made osteotomy cutting guide (Fig. 3), inserted on the three guide wires, the first, 2-cm vertical osteotomy is performed on a coronal plane, posteriorly to the tibial tuberosity, through an oscillating bone saw and completed with a sharp thin osteotome without violating the lateral cortex (Fig. 4). The second, 1-cm horizontal osteotomy is performed on a oblique plane just above the tibial tuberosity behind the patellar tendon, oriented at approximately 120° with respect to and starting at the proximal end of the first coronal osteotomy (Fig. 5). This second osteotomy is completed, including the lateral cortex of the tibial tubercle, using a thin 1-cm large osteotome.

The third final horizontal osteotomy involving 70% of the tibial width starts from the posterior aspect of the tibia. The osteotomy is performed in posteromedial–anterolateral direction, just below the three guide wires, using an oscillating saw and carefully completed with a thin sharp 2-cm large osteotome. The oblique upward direction plane of this last osteotomy is parallel and in strict contact with the three guide wires to prevent its accidental proximal migration into the joint. The osteotomy is completed without affecting the lateral cortex and preserving the anterior cortex and tibial tubercle.

**Fig. 6:**
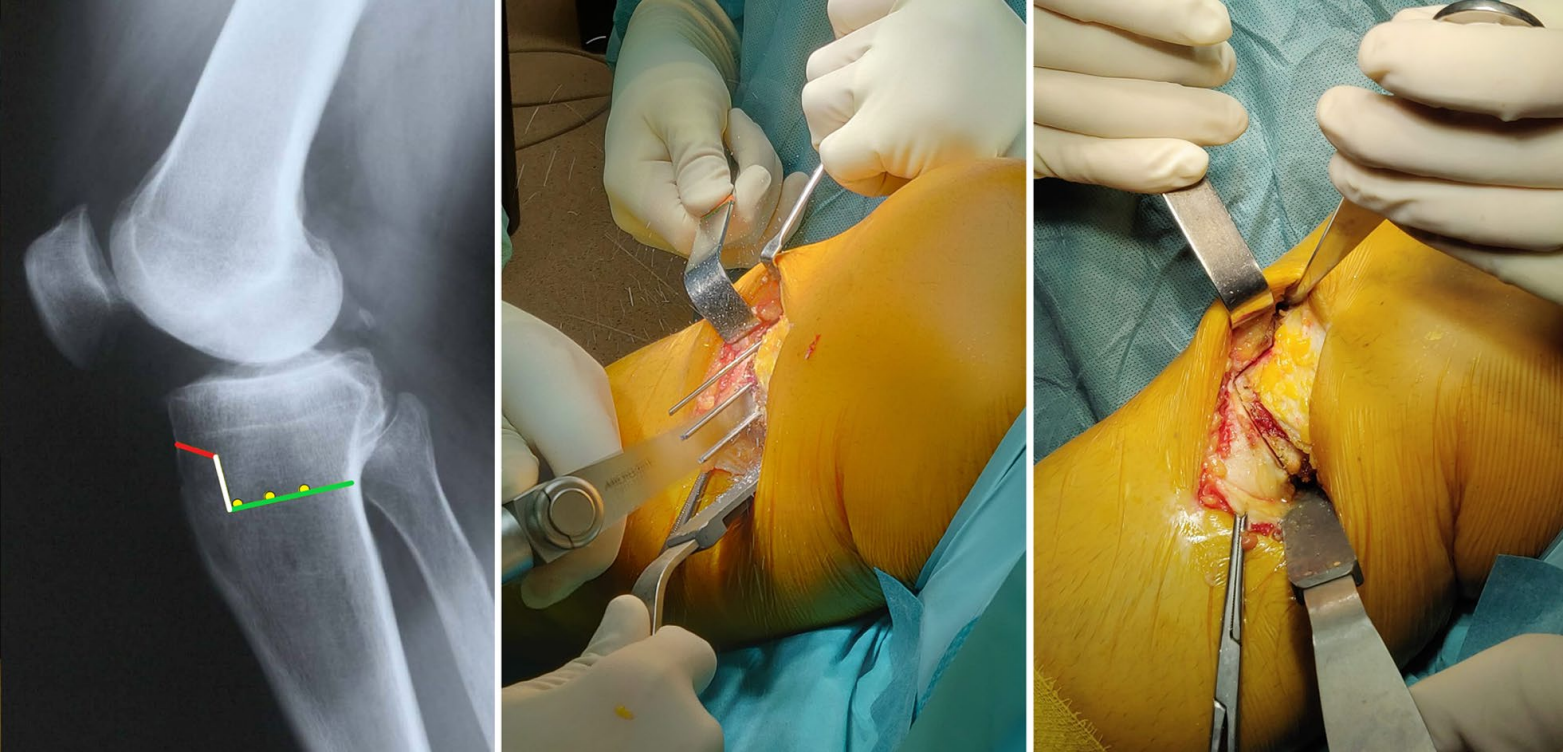
On the left, a graphical representation of the three guide wires (yellow dots), the first coronal osteotomy (vertical white line), the second oblique osteotomy (red line), and the third horizontal main osteotomy, on a lateral X-ray view of a right knee. In the middle, intraoperative findings of the third horizontal osteotomy performed with an oscillating saw. On the right, the intraoperative findings of complete Z-shaped MOWHTO, in a right tibia, from medial view

The complete osteotomy should appear Z-shaped in a sagittal plane, making certain that all the cancellous metaphysis and, especially, only the posterior cortex is completely interrupted, preserving a lateral hinge of about 1 cm of intact bone (Fig. 6).

**Fig. 7:**
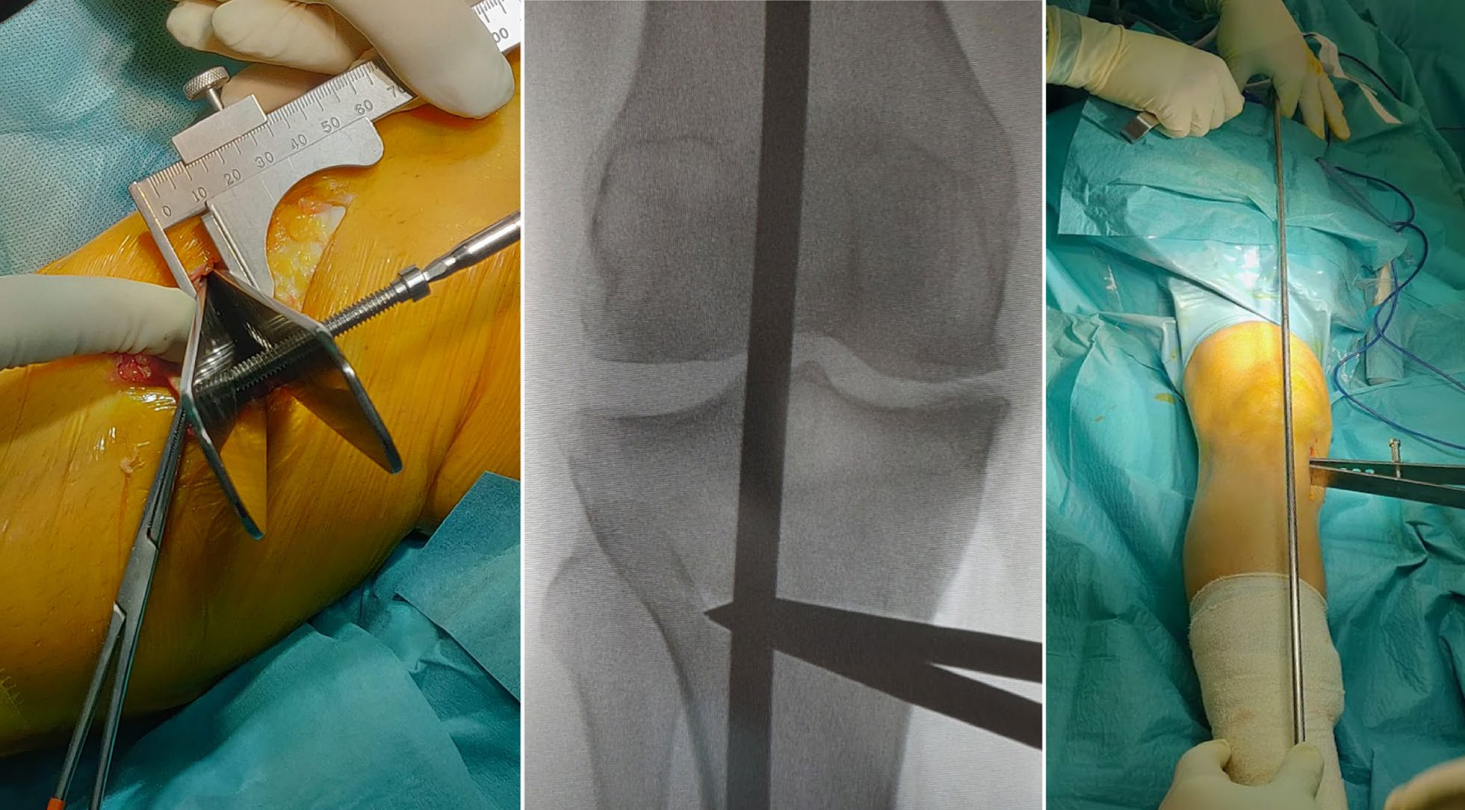
On the left, intraoperative findings of the distractor positioned and gradually opened into the major horizontal osteotomy site, on a right tibia from medial view. In the middle, the intraoperative alignment fluoroscopic control, verified through the rod guide of a right knee. On the right, the intraoperative finding of the varus correction of the right lower limb, through the distractor and verified through the rod guide

**Fig. 8:**
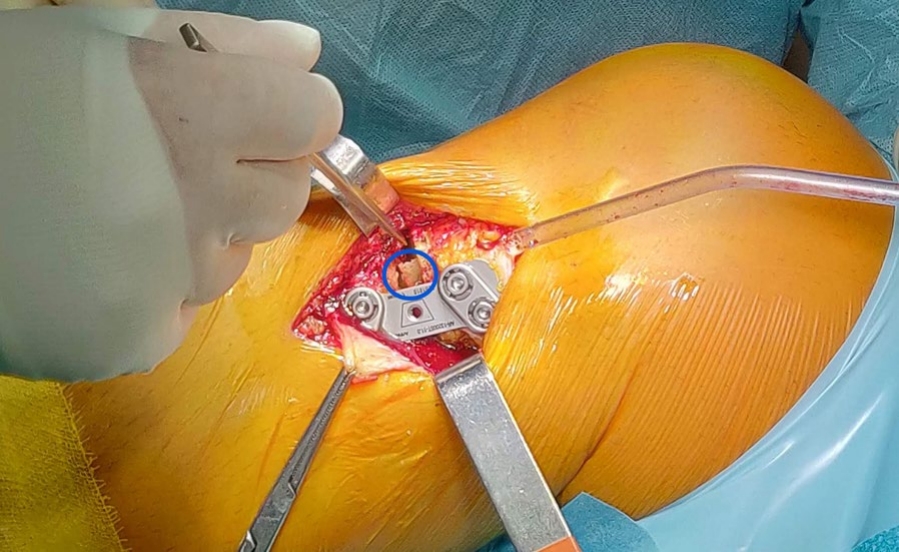
Intraoperative finding of the Puddu plate positioned and fixed on the posteomedial aspect of the main horizontal osteotomy site, from medial view. The cancellous bone bract detached from both the proximal and distal sides of the site of osteotomy to create a bridge of bone contact that favors healing of the fracture can be seen in the circle

Then, a distractor is inserted into the major oblique osteotomy site to gradually open the gap until adequate correction is achieved. The correct alignment restoration is verified under fluoroscopy, through a guide rod, placed from the center of the femoral head to the center of the talus. On the image intensifier centered on the knee, the guide rod should lie just lateral to lateral tibial spine, leaving 62.5% of the tibial plate width medially (Fig. 7). Alignment correction is possible by opening or closing the gap according to the preoperative planning and under direct fluoroscopic control. The fixation of the osteotomy is achieved using the Tibia Opening Wedge Osteotomy Plate (Arthrex Inc., Naples, FL, USA), also known as the “Puddu plate.” The plate is positioned on the medial cortex of the tibia as posteriorly as possible and fixed proximally with two cancellous screws and distally with two cortical screws (Fig. 8). The osteotomy gap is not filled with bone graft or bone substitute; only a local bridge of free autologous cancellous bone, between the proximal and the distal side of the osteotomy, is developed using a standard curette in the wider medial portion of the gap to promote healing (Fig. 8). Final fluoroscopic assessment ensures adequate positioning of the hardware. After the implant of the Puddu plate the pes anserinus is reinserted, suction drain is placed, and the wound is closed in layered fashion.

### Postoperative aftercare

Antithrombotic prophylaxis (low-molecular-weight heparin) was administered for 40 days in all patients, starting from the first postoperative day. Drains were removed on the second postoperative day, after which walking with two crutches without weight-bearing was allowed. The rehabilitation program included a long-leg hinged brace for 4 weeks. Limited continuous passive motion (CPM; 0–40°) was begun on the first postoperative day, and 90° knee flexion was achieved on the third postoperative day, with the assistance of a physiotherapist. Partial weight-bearing began 40 days after surgery, and full weight-bearing after 60 days.

### Clinical assessment

Patients were evaluated preoperatively then at last follow-up by the same surgeon who performed the procedure.

All measurements and complications (intraoperative fracture, persistent pain, delayed union or nonunion, infection, and thromboembolism) were recorded.

Clinical evaluation of knee pain was measured using a self-administered 100-mm visual analog scale (VAS) (0 mm, no pain; 100 mm, worst pain) [32]. Pain was evaluated upon walking. Functional outcomes were assessed by WOMAC [33].

At last follow-up, patients were asked about their satisfaction with the surgery in general. The responses were graded on a five-point Likert scale from “totally satisfied” to “very dissatisfied” [34]. All the clinical data were recorded by the senior surgeon (M.P.) in his personal digital database (VisualMed by Efeso Active Marketing S.r.l).

### Radiographic assessment

Standard anteroposterior and lateral view radiographs of the affected knee were performed on the first day postoperatively then at 1, 3, 6, and 12 months postoperatively. At last follow-up, all the patients also underwent a full-length standing anteroposterior radiograph of the entire lower extremities. Radiographic analysis was performed by the same independent assessor (orthopedic surgeon) to determine the following: tibiofemoral osteoarthritis grade in the Ahlbäck classification system [31], tibial slope (TS) using the angle between the joint line and the perpendicular to the posterior tibial cortex line [35], Caton–Deschamps index (CD) of patellar height [36], and degree of bone healing.

Correction in the frontal plane was evaluated by comparing the medial proximal tibial angle (MPTA), preoperatively and on the follow-up radiographs [37]. MPTA was defined as the angle between the proximal anatomical axis of the tibia and a tangent along the articular surface of the tibial plateau and can be used to predict the varus degree correction [38].

### Statistical analyses

Statistical analysis was carried out using SPSS for Windows 16.0 (SPSS, Inc., Chicago, IL). Shapiro–Wilk test was used to evaluate the normality of distributions of variables. Paired data (preoperative versus postoperative values) that passed the Shapiro–Wilk test for normality were compared using Student’s *t*-test, while nonparametric variables were analyzed using Wilcoxon’s test. The Kaplan–Meier method was applied to assess data that were considered censored, namely survival and revision surgery. *P* < 0.05 was considered significant.

## Results

### Enrollment process

The principal independent assessor (P.P.) identified and enrolled, according to the eligibility criteria, 75 patients (80 knees) who underwent Z-shaped MOWHTO from January 2001. All the data were retrieved from the senior surgeon’s (M.P.) personal digital database (VisualMed by Efeso Active Marketing S.r.l). Data were not available for five (6.7%) patients who were considered lost at final follow-up and one patient (1.3%) who died (Table 1).

**Table 1:**
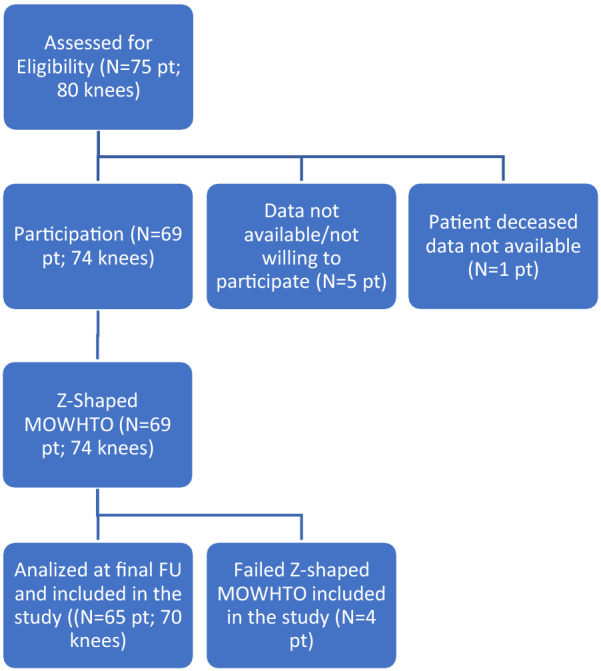
Enrollment process flow diagram

### General results and demographic analysis

The study included 80 knees of 75 patients, 54 male and 21 female, with mean age of 45.8 years (range 29–62 years). Mean preoperative body mass index (BMI) was 27.3 ± 3.4 kg/m^2^ (Table 2).

**Table 2:** Demographic analysis at baseline

Patient characteristics (*N* = 75 patients, 80 knees)	
Gender	
Male (%)	54 (72%)
Female (%)	21 (28%)
Mean (range) age (years)	45.8 (29–62)
Mean ± SD BMI (kg/m^2^)	27.3 ± 3.4

Patients’ demographic data before surgery. The study included, at baseline, 75 patients for a total of 80 knees. Gender is reported in absolute values and percentage

After median follow-up of 7.2 (95% CI 5.6–9.2) years, 74 knees in 69 patients were assessed based on an in-person visit and imaging studies.

Z-shaped MOWHTO failed in four knees, after a mean of 9.25 ± 1.86 years. In all four cases, total knee arthroplasty was performed because of recurrence of symptoms and radiological progression of osteoarthritis (MPTA values at revision surgery: 84.5°, 83.6°, 85.2°, and 82.8°, respectively).

**Fig. 9:**
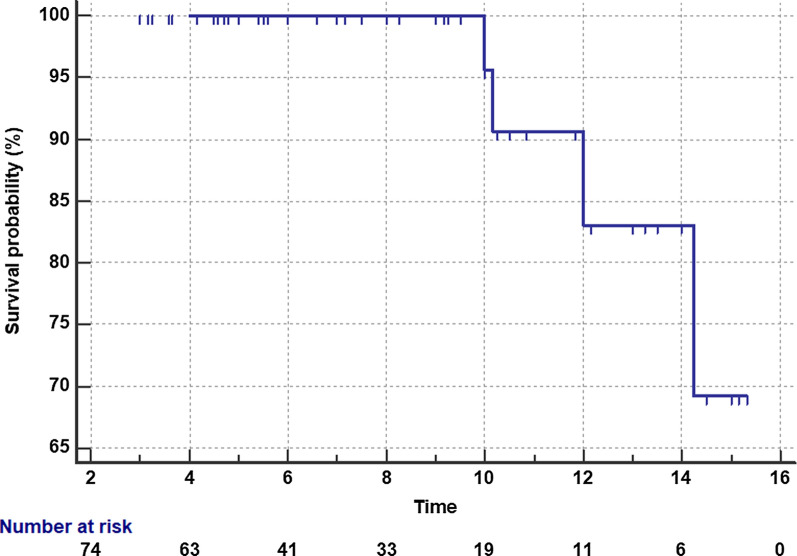
Kaplan–Meier (K–M) survival plot. The K–M estimator analysis showed an overall survival rate at 10 years of 95 ± 1.7%

Kaplan–Meier analysis showed an overall survival rate at 10 years of 95 ± 1.7%. Only 33 knees (44,6%) were assessed beyond the median follow-up time of this study, at 8 years after surgery, showing a survival rate of 100% (Fig. 9).

### Complications

No patients were affected by any of the following complications: lateral tibial cortex or intraarticular fracture, deep venous thrombosis, pulmonary emboli, superficial or deep infection, neurovascular injuries, delayed union or nonunion, or delay in rehabilitation program. One plate and screw rupture was observed before removal.

### Clinical outcomes

The detailed results of the clinical scores (VAS, WOMAC) are presented in Table 3. Statistically significant improvements (*P* < 0.001) of both scores compared with preoperatively were observed at final follow-up.

**Table 3:** Clinical and radiographic outcomes

	Preoperative	Last follow-up	*P* value
VAS	5.32 ± 0.7	0.9 ± 0.9	< 0.001
WOMAC	50.1 ± 11.9	12.7 ± 8.1	< 0.001
MPTA	83.8 ± 1.2°	90.5 ± 1.1°	< 0.001
TS	4.1 ± 2.9°	4.5 ± 3.1°	0.187
CD index	0.90 ± 0.14	0.76 ± 0.11	< 0.001

*VAS* visual analog scale, *WOMAC* Western Ontario McMaster University Osteoarthritis Index, *MPTA* medial proximal tibial angle, *TS* tibial slope, *CD* Caton–Deschamps

Most of the patients were satisfied (“totally satisfied” 58%, “fairly satisfied” 26%, or “slightly satisfied” 13%) with the surgery at last follow-up, while only two patients were “slightly dissatisfied” (3%).

### Radiological outcomes

The mean MPTA changed significantly from 83.8 ± 1.2° preoperatively (range 81.6–86.5°) to 90.5 ± 1.1° at last follow-up (range 89–93.5°) (*P* < 0.001). The mean correction angle was 6.7 ± 1°.

The TS increased, by a mean of 0.4° (*P* = 0.187). The Caton–Deschamps index decreased significantly, by a mean of 0.14 (*P* < 0.001) (Table 3).

**Fig. 10:**
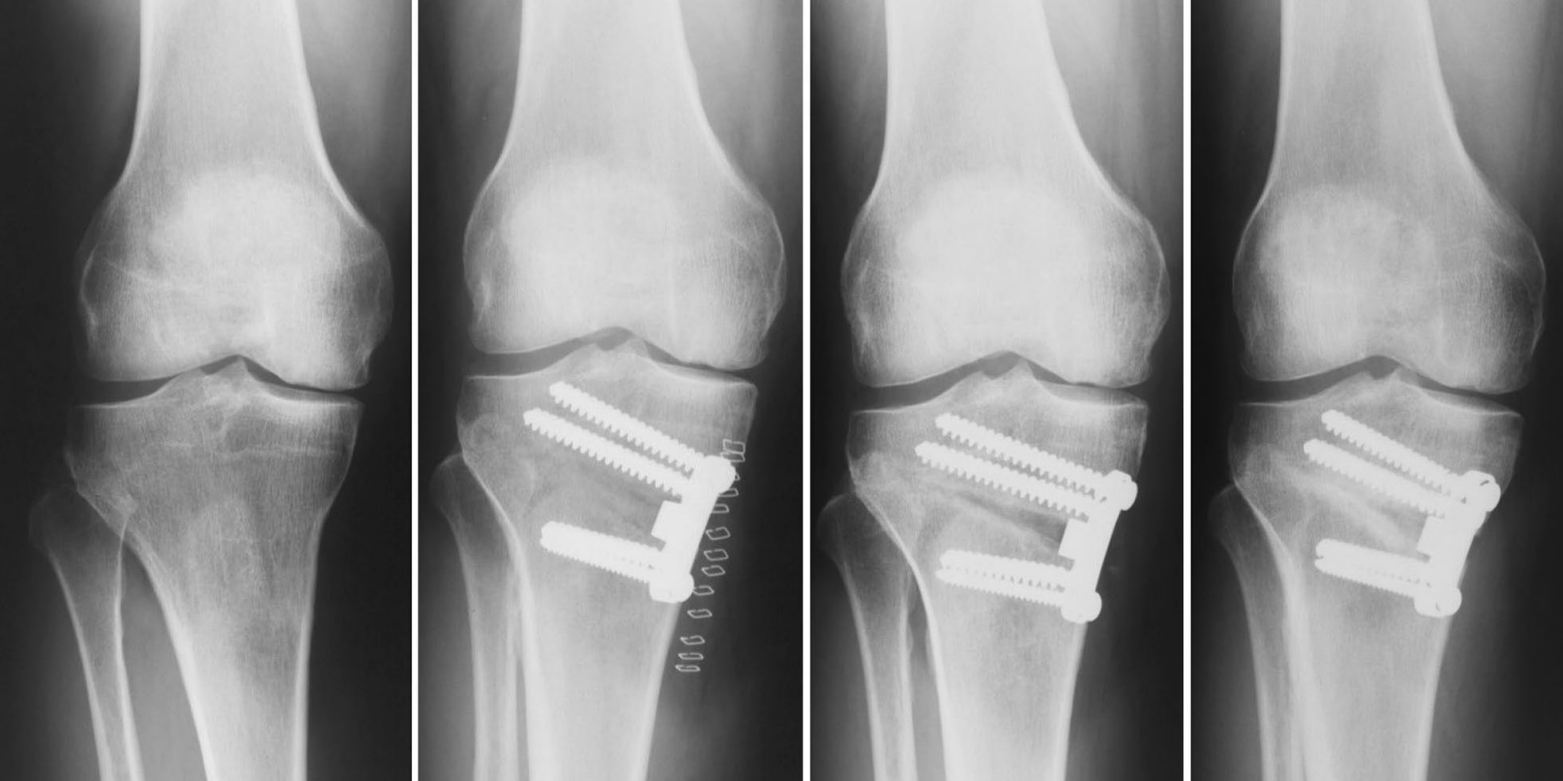
Anteroposterior X-ray views of the same right knee. From left to right, respectively: preoperatively, and 1 day, 1 month, and 3 months postoperatively

Mean time to bone healing was 12 weeks, defined as full osteotomy gap filled with new bone (Fig. 10). No relationship was found between the difference in MPTA compared with change in tibial slope, BMI, or age.

## Discussion

This retrospective series of 74 knees treated with MOWHTO using a novel Z-shaped osteotomy showed good results for both clinical and radiological outcomes at 7.2 years follow-up.

These results are similar to outcomes of biplanar osteotomy previously reported [4–6]. Overall, the MPTA was slightly overcorrected, compared with the normal range (85–90°) [18], although the degree of overcorrection achieved in these patients is considered clinically irrelevant [39].

Tibial slope did not increase significantly. The posterior tibial slope increase can be prevented with a simple distraction at the most posterior aspect of the osteotomy gap and with the subsequent positioning and fixation of the plate slightly posterior to the tibial axis [21, 24, 25]. Equal anterior and posterior medial gaps might increase the tibial slope because of the anatomic characteristics of the proximal tibia [19, 40].

Conversely, the Caton–Deschamps index decreased significantly postoperatively, but as highlighted in a recent review by Mingliang et al., this change does not affect short-term patient satisfaction [13].

In terms of clinical evaluation, significant improvements in the VAS and WOMAC scores were observed. The positive correlation between the preoperative and final VAS and WOMAC scores shows that clinical scores improved when pain decreased.

In this study, we report only one case of delayed healing, in a heavy smoker. Smoking and overweight/obesity play a major role in development of nonunion in patients treated with HTO [41].

No fracture of the lateral cortex was reported. The incidence of fractures in MOWHTO is significantly higher than in lateral closing-wedge HTO [13]. This complication may lead to instability at the osteotomy site, delayed union, nonunion, and recurrent varus [26]. A meticulous surgical procedure is a critical step to decrease the incidence of lateral cortical hinge fracture. Lateral cortical fracture during MOWHTO has been significantly associated with wedge size and opening distance [42, 43]. The risk of lateral cortex fracture is correlated with osteotomy angle amplitude, even reaching 90% when the correction angle is higher than 8° [44–46]. The biplanar biomechanical properties of MOWHTO compared with uniplanar MOWHTO allow the application of higher opening-wedge load and the achievement of larger correction degrees before encountering lateral cortex fracture [42]. Therefore, because of its effective reduction of the incidence of lateral cortical fracture, biplanar MOWHTO should be preferred over uniplanar MOWHTO [13, 47].

Furthermore, biplanar MOWHTO produces smaller volumes of “empty gaps” combined with larger cancellous bone surface in contact, compared with the uniplanar MOWHTO technique [47]. Literature describes two healing processes occurring simultaneously after MOWHTO: primary bone healing between the bone surfaces in contact, and secondary bone healing within the gap [13, 47, 48]. Since contact healing is faster than gap healing under stable conditions, the geometrical conformation of biplanar MOWHTO theoretically promotes osteotomy healing, allowing wider bone contact surfaces [47]. This could also explain the good efficacy of rapid bone healing in our series with the exclusive use of local bone autograft without the need to fill the gap with bone harvested from iliac crest or synthetic substitutes [49]. Goshima et al. report that, in around 60% of the “V-shaped” biplanar MOWHTOs performed in their study, only 25% of the volume of the osteotomy gap was filled 3 months postoperatively [50]. In contrast, Staubli et al. [51] reported that, on standard radiographs, at least 75% of the gap was filled with new bone within 6−18 months.

In this study, the average osteotomy healing time was 3 months. We thus deduce that Z-shaped biplanar, rather than V-shaped biplanar MOWHTO, better reflects the geometrical principles required for bone healing, providing an even larger cancellous bone surface combined with a smaller wedge gap volume. Thus, this newly described open-wedge Z-shaped osteotomy may allow the creation of a larger gap, maintaining larger bone surface contact areas, compared with the “classic,” V-shaped biplanar technique and uniplanar techniques.

Pape et al. demonstrated in a cadaveric study that biplanar osteotomy significantly increased the fixation stability in anteroposterior and rotational planes, especially when short spacer plates, such as the Puddu plate, are used [52]. The additional biomechanical stabilizing effect of V-shaped biplanar MOWHTO and the biologic bone healing improvement reported are both potentially enhanced by the geometric structure of the Z-shaped biplanar MOWHTO presented.

The long-term survivorship is higher after MOWHTO than lateral-closing wedge HTO (LCWHTO), with lower fracture rate [13]. In a recent systematic review, Mingliang et al. reported a pooled 10-year survival rate that was 6.2% greater for MOWHTO than LCWHTO [13]. In this study, the survival rate at median follow-up of 7.2 years after Z-shaped MOWHTO was 95 ± 1.7%, in line with the survival rate presented in literature for MOWHTO (91.6%) [13].

Currently, the most common surgical interventions performed for the treatment of medial osteoarthritis (OA) of the knee are MOWHTO and medial unicompartmental knee arthroplasty (UKA). To the best of the authors’ knowledge, a wide literature suggests that good outcomes can be achieved after either procedure. A recent systematic review investigating the return to physical activity after HTO or UKA of 2023 pooled patients showed equal or improved scores, for activity and knee function, regardless of the operation performed [53]. Traditional indications for HTO, such as younger age and BMI < 30 kg/m^2^, were confirmed in that systematic review [53]. The authors reported that the HTO group was 12.2 years younger than the UKA group, with mean age at surgery of 48.4 and 60.6 years, for the HTO and UKA group, respectively. Their findings are in line with the mean baseline age of the patients in the present study (45.8 years) [53]. A Finnish registry-based study on survivorship after 3195 HTOs clearly showed that patients aged less than 50 years had significantly lower risk of conversion to TKR than older people [54]. This allows us to speculate that, despite the similar clinical results of the two procedures, if patients undergo UKA at younger age than indicated, attention must be paid to the subsequent risk of revision to total knee arthroplasty (TKA) [53]. A previous metaanalysis reported a mean revision time to TKA, after UKA, of 8.2 years after surgery, but 9.7 years after HTO [55]. Furthermore, considering that TKA after UKA showed an increased risk of undergoing “re-revision,” when compared with TKA after HTO, we emphasize that caution should be taken when UKA is offered to patients younger than 60 years old [56].

There are several limitations to this study.

The first limitation is reproducibility. The self-made guides allow the performance of the osteotomies safely, precisely, and easily, but these devices are essential to this technique. A standardized multicentric study should further examine the surgical learning curve and better evaluate intraoperative complications. Although data were recorded prospectively, this is a retrospective outcome evaluation of surgeries performed by a single senior surgeon. The loss of MPTA angle correction over time (difference between the early postoperative value and the value at last follow-up) was not assessed. Another limitation consists in the wide variability of age among the patients. This may have led to some bias in the results presented.

Furthermore, since many heterogeneous methods have been described to assess osteotomy healing on radiographs, and since X-rays do not usually even provide an adequate representation of the osteotomy gap and thus ossification, we decided to define the osteotomy as healed only when we could see the gap completely filled. This methodological flaw made it difficult to compare the gap filling timing with results reported in literature.

Finally, even if this study benefits from clinical and radiographic evaluation at 7.2 years of follow-up, performed by an independent assessor, MOWHTO outcomes could deteriorate over time, particularly after 10–15 years, requiring longer follow-up to provide a definitive conclusion on this technique [18, 57].

## Conclusions

With a survival rate of over 95% at 7.2 years follow-up, MWHTO with a Z-shaped technique seems to be a reliable treatment option for managing medial knee compartment osteoarthritis with varus malalignment in younger active patients. Modified biplanar Z-shaped MOWHTO is a safe and reliable technique that offers satisfactory clinical and radiological medium-term outcomes. The distinctive three-dimensional geometrical conformation provides a theoretically better environment for bone healing, increased anteroposterior and rotational stability, and safer opening-wedge loading force application with a low lateral hinge fracture risk. To better assess the advantages of this Z-shaped biplanar MOWHTO, in terms of osteotomy healing time, further studies, with higher level of evidence, are needed.

Furthermore, although the TKA conversion rate after Z-shaped MOWHTO is low, as emerged from this case series, studies with longer follow-up are required to further evaluate this novel technique.

## Data Availability

The datasets used and/or analyzed during the current study are available from the corresponding author on reasonable request.

## Abbreviations

OA: Osteoarthritis
TKA: Total knee arthroplasty
HTO: High tibial osteotomy
LCWHTO: Lateral closing-wedge high tibial osteotomy
MOWHTO: Medial opening-wedge high tibial osteotomy
BMI: Body mass index
CPM: Continuous passive motion
VAS: Visual analogue scale
WOMAC: Western Ontario and McMaster University Osteoarthritis Index
TS: Tibial slope
CD: Caton–Deschamps index
MPTA: Medial proximal tibial angle
